# Multiscale variations of the crustal stress field throughout North America

**DOI:** 10.1038/s41467-020-15841-5

**Published:** 2020-04-23

**Authors:** Jens-Erik Lund Snee, Mark D. Zoback

**Affiliations:** 1 Department of Geophysics, Stanford University, 397 Panama Mall, Mitchell Bld. 3rd Flr., Stanford, CA 94305 USA; 2 Present Address: Geosciences and Environmental Change Science Center, U.S. Geological Survey, P.O. Box 25046, MS 980, Denver, CO 25046 USA

**Keywords:** Geodynamics, Geophysics, Tectonics

## Abstract

The Earth’s crustal stress field controls active deformation and reflects the processes driving plate tectonics. Here we present the first quantitative synthesis of relative principal stress magnitudes throughout North America together with hundreds of new horizontal stress orientations, revealing coherent stress fields at various scales. A continent-scale transition from compression (strike-slip and/or reverse faulting) in eastern North America to strike-slip faulting in the mid-continent to predominantly extension in western intraplate North America is likely due (at least in part) to drag at the base of the lithosphere. Published geodynamic models, incorporating gravitational potential energy and tractions from plate motions or relative mantle flow, successfully predict most large-wavelength stress rotations but not the shorter-wavelength (<~200 km) rotations observed in the western USA. The stresses resulting from glacial isostatic adjustment appear to be much smaller than the magnitude of ambient tectonic stresses in the crust at depth.

## Abstract (web summary)

The authors here present a stress map of the North American crust that gives a new view of dynamics of the continent. The results can be applied to probabilistic seismic hazard analysis and resource development as well as to provide constraints for theoretical models of crustal dynamics.

## Introduction

Knowledge of horizontal principal stress directions and relative stress magnitudes is fundamental to understanding the mechanical behavior of the Earth’s crust, including the factors driving plate motion and seismicity. Recent studies have debated what proportion of intraplate deformation and topography is attributable to forces from plate boundary interactions, gravitational potential energy (GPE) within the lithosphere, and mantle flow^1–5^. Initial plate-scale stress mapping identified provinces of relatively constant stress separated by transition zones where stress orientations could rotate rapidly^6^. Until recently, these studies were limited by a lack of quantitative constraints on relative principal stress magnitudes and large gaps in knowledge of horizontal principal stress orientations. This lack of constraints has created challenges for predicting the style of earthquakes in many areas, and for validating models of crustal dynamics, such as those predicting stress and seismicity owing to postglacial rebound^7,8^. In North America, recent efforts to map both *S*_Hmax_ orientations and relative stress magnitudes have been limited to regional scales^9–13^. Unfortunately, some recent studies appear to have involved inversions of apparently unreliable focal plane mechanisms^14^, yielding *S*_Hmax_ orientations (and relative stress magnitudes) that are frequently in conflict with one another as well as the state of stress documented in previous studies^11–13,15–17^.

**Fig. 1:**
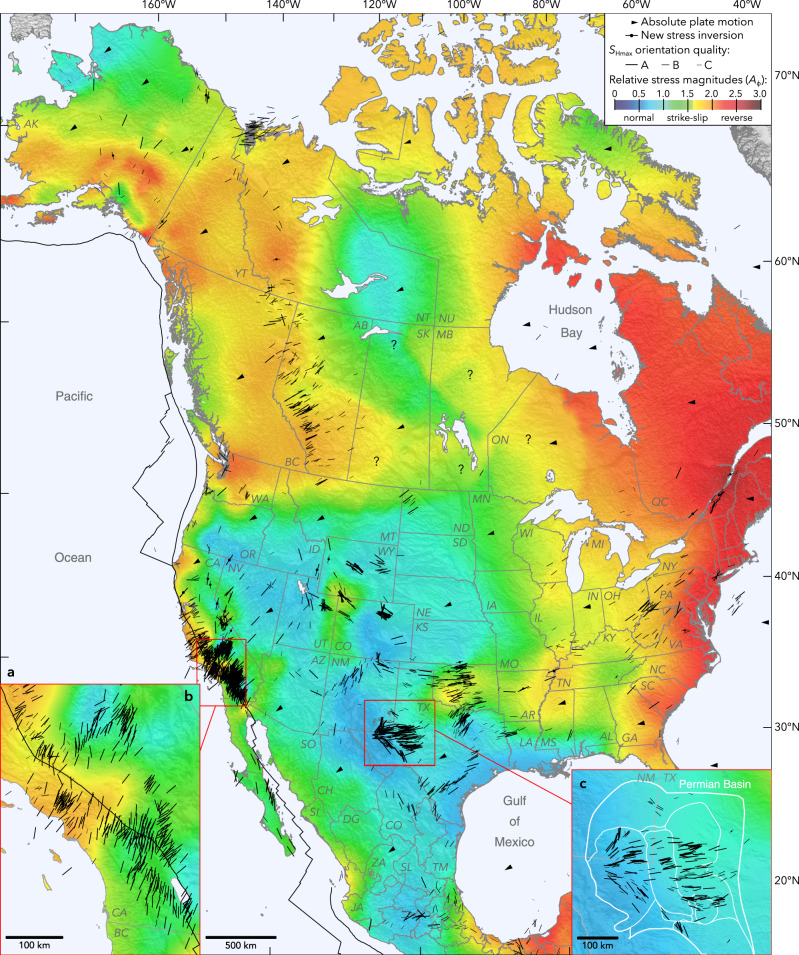
State of stress in North America. Black lines are maximum horizontal stress (*S*_Hmax_) orientations and the colored background is the style of faulting (relative stress magnitudes) compiled from new and previously published measurements (see the text and Supplementary Information for data sources). *S*_Hmax_ orientations span depths throughout the brittle upper crust (Supplementary Fig. 6). Relative stress magnitudes are classified based on the *A*_ϕ_ stress classification system of Simpson^57^ (see text and legend) and were interpolated (and slightly smoothed) from almost 2000 individual measurements. NNR-MORVEL56 absolute plate motion directions (as indicated by the pointed arrowheads) are from Argus et al.^30^.

We have constructed a new-generation stress map of North America (Fig. 1) that includes >300 new stress orientations and a systematic mapping of relative stress magnitudes using well-constrained earthquake focal mechanisms and sense of recent fault slip at almost 2000 locations throughout North America. The new map confirms both the consistency among the different types of upper crustal stress indicators (and quality control criteria) utilized by prior workers^6,16,18^ as well as continent-scale trends in maximum horizontal principal stress (*S*_Hmax_) orientations. Moreover, we provide here far more detailed data in many areas, revealing coherent variations of both stress orientation and relative stress magnitudes at multiple scales. In this paper, we discuss the newly seen patterns of stress variations in the context of the possibly causative geologic processes, and we compare the observations with predictions from geodynamic models that consider the effects of plate boundary tractions, basal drag owing to modeled mantle flow, and variations in GPE.

## Results and discussion

### Variability in the stress field and sources of stress

At the continent scale, the stress field is principally compressive: strike-slip/reverse and reverse faulting (RF) in eastern parts of North America and broadly strike-slip (SS) faulting in the center of the continent. Normal faulting (NF) is seen in the Basin and Range province, Rio Grande Rift, and the western Great Plains. Extensional stress fields are also seen in northeast Texas, sedimentary rocks of the Gulf Coast, and in parts of Mexico and Alaska. Plate interactions are especially apparent in a narrow zone along the western margin that is subject to varying components of convergence and shear traction. The high-resolution picture of the stress field now available in many regions documents local and regional variability that is especially pronounced in parts of the western USA, as discussed below. In some cases, these variations allow us to identify sources of regional and local stress.

Lateral variations in GPE represent another source of intraplate stress variability. In general, locally elevated GPE superimposes a component of horizontal extensional stress, and this outward spreading also imposes compression in adjacent regions^19,20^. Because GPE can be simply defined as the integral of density perturbation times gravity from the surface to a certain depth^21^, it depends at a given depth upon surface elevation and vertical variations in density (and a second-order term from self-gravity). For isostatically compensated lithosphere, static sources of GPE anomalies include inhomogeneities of crustal thickness (thicker crust may be buoyant, increasing GPE^19^), lithospheric density (higher density can pull the crust downward and thereby reduce GPE^22^), and lithospheric thickness (thicker subcrustal lithospheric roots are typically thermally dense and can reduce GPE by pulling lithosphere downward^23^). (Very old cratonal lithosphere may be chemically buoyant, which offsets the thermal effect^24^.) Because thicker and/or less-dense crust will produce elevated topography under isostatically compensated conditions, higher topography is often associated with higher GPE. In areas experiencing ongoing postglacial rebound, GPE will be lower in the depressed zone beneath the former ice sheet, imposing a component of compression in the brittle upper crust^25^. Tractions on the lithosphere applied by radial flow of mantle material represents another dynamic source of GPE, which may perturb surface elevation (dynamic topography) and can affect the stress field^21^.

**Fig. 2:**
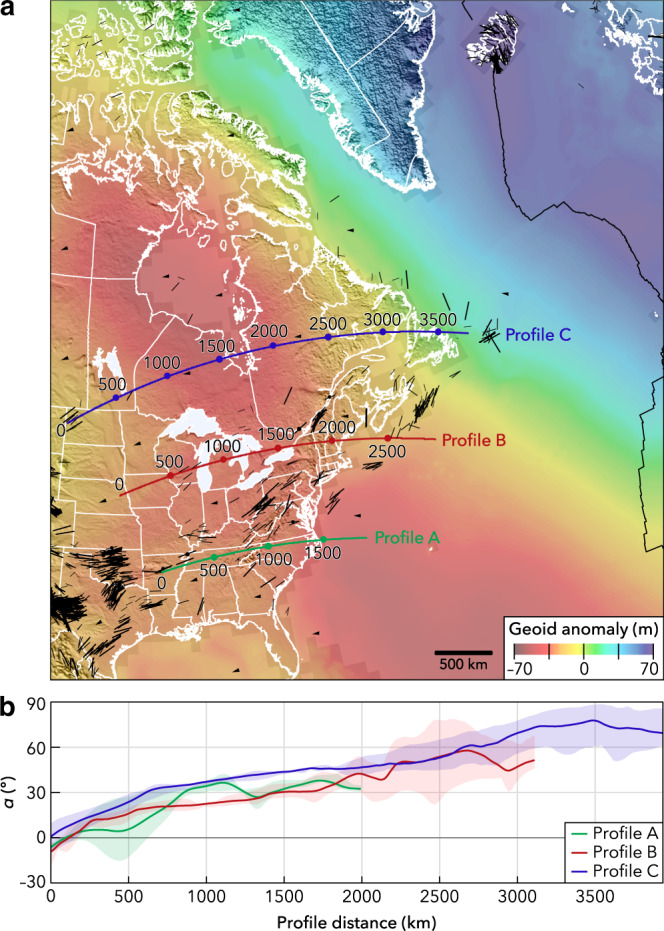
Relationship between absolute plate motion directions and maximum horizontal stress (*S*_Hmax_) orientations in central and eastern North America. **a** Stress map subset with profiles parallel to absolute plate motion. **b** Angle *α*, the mean divergence between *S*_Hmax_ orientations and plate motion, was sampled every 0.1° along 4°-wide swath profiles from a slightly smoothed grid of divergence angles. The shaded regions represent the full range of divergence angles sampled across each swath profile. Divergence angles were estimated by subtracting interpolated A and B quality *S*_Hmax_ orientations from NNR-MORVEL56^30^ plate motions.

**Fig. 3:**
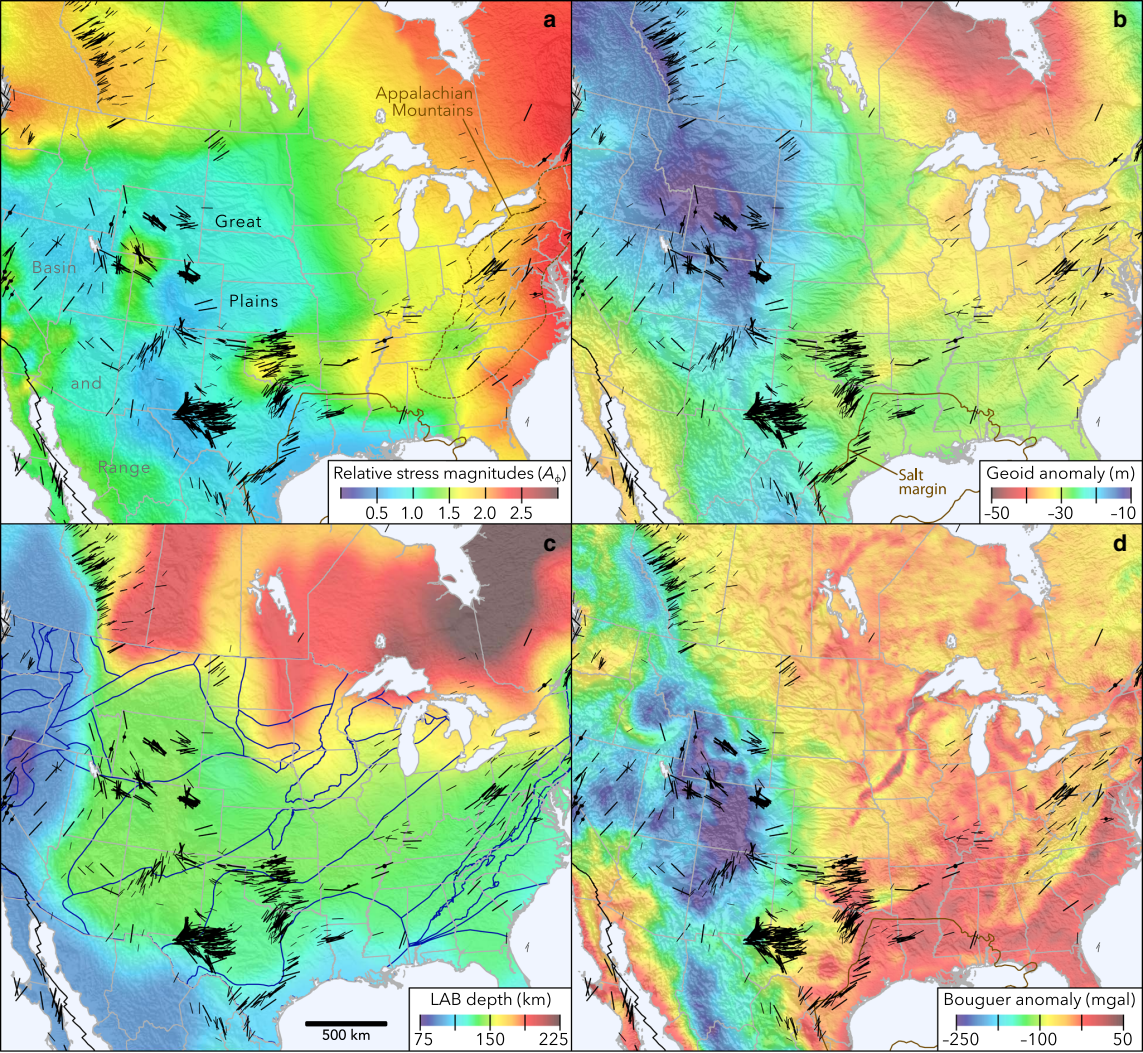
Comparison between the state of stress and lithospheric data. **a** State of stress in the central and eastern USA and southeast Canada. For clarity, dense maximum horizontal stress (*S*_Hmax_) orientations in southern California are omitted. The Gulf of Mexico salt margin is from Pindell and Kennan^61^. **b** Geoid anomaly from Pavlis et al.^62^. **c** Lithosphere–asthenosphere boundary (LAB) depth from Artemieva^38^. Blue lines are crustal basement domain boundaries from Lund et al.^40^. **d** Bouguer gravity anomaly from Mooney and Kaban^29^.

As shown in Fig. 2a, *S*_Hmax_ is ~ENE–WSW in the central USA, subparallel to absolute plate motion, which could suggest that the compression direction is related in some places to the motion of North American lithosphere over the asthenosphere. However, progressing eastward *S*_Hmax_ diverges from the plate trajectory at a similar rate along all three profiles, and it is clearly quite oblique toward the east side of the continent and offshore continental margin, especially offshore eastern Canada (Fig. 2b). The cause of this divergence is not understood but suggests that the ridge-push force^26^ is not the dominant contributor to the stress field along the eastern margin of North America. On the continental margin, some of the divergence could result from a superimposed component of margin-normal extension owing to sediment loading and resulting flexure in the brittle crust, which would rotate *S*_Hmax_ subparallel to the margin, potentially without producing an extensional stress field^18^. Especially in eastern Canada (Profile C), the orientation of *S*_Hmax_ very oblique to plate motion may also be related to compression imposed adjacent to a broad topographic swell and strongly positive geoid anomaly in the north Atlantic associated with the Mid-Atlantic Ridge and Icelandic hotspot (Fig. 2a), which is possibly supported by a mantle plume^20^. Where *S*_Hmax_ deviates from plate motion directions within the eastern USA (Profile A), it is subparallel to the trend of the Appalachian orogen and crustal-scale faults^27^. The Appalachians are underlain by a belt of thick crust that is likely more buoyant than the crust in adjacent areas, as indicated by a pronounced Bouguer gravity anomaly low (Fig. 3d) that coincides with much of the mountain range^28,29^, indicating potentially low GPE that would apply extension perpendicular to its axis and could rotate *S*_Hmax_ toward the orogenic trend. Imposed extensional stresses owing to buoyancy of Appalachian crust (together with higher geoid anomaly south of Kentucky) could also explain an anomalous zone of strike-slip faulting (lower compressive stresses than surrounding areas) in Alabama and eastern Tennessee.

On the basis of sparser data, previous workers argued against the movement of the North American plate being caused by a significant driving traction associated with relative motion of the viscous asthenosphere^6^. The data we present here delineate a first-order stress gradient across central and eastern North America, indicating a long-wavelength reduction of horizontal compressive stress. To obey the equations of equilibrium, this gradient must be balanced by an opposing shear stress, which at this scale (and with the gradient oriented in the direction of plate motion^30^) is likely imposed by basal drag^31,32^.

East of the Appalachians, a zone of thinner (~32–40 km) crust^33^ (and higher Bouguer gravity anomaly) may be partly responsible for reverse faulting along the Eastern Seaboard. Despite this and the Appalachian example, it is noteworthy that variations in crustal thickness are generally not well correlated with faulting regime elsewhere in North America. In several areas, changes in *A*_ϕ_ are in fact opposite what would be expected due to variations in crustal thickness (e.g., thinner crust under the extensional northern Basin and Range, western USA, and thicker crust under areas of SS/RF in southwest Oklahoma), indicating stronger effects from other factors, especially lithospheric thickness and crustal GPE.

Geoid anomalies serve as a useful approximation of GPE where the lithosphere is in isostatic equilibrium^34^. As shown in Figs. 3a and 3b, the geoid anomaly is strongly correlated with relative stress magnitudes in central and eastern North America. Where the geoid anomaly is lower, the faulting regime is more compressive, and vice versa, as is expected. These effects are partly related to deglaciation. The strongly negative geoid anomaly centered near Hudson Bay indicates negative GPE (and a likely source of compressive stress for this region) associated with downwarped lithosphere that continues to rebound following melting of the Laurentide ice sheet^25^. Below eastern North America, mass from the subducted Farallon slab probably increases the geoid anomaly^35^. Additional mass below the compensation depth violates the assumption of isostatically compensated masses that is the basis of the inverse scaling relationship between geoid anomaly and stress magnitudes. Nevertheless, subtracting away any added geoid anomaly owing to the deep slab would simply amplify the inverse relationship that we observe between geoid and relative stress magnitudes in eastern North America.

In certain areas, there are pronounced exceptions to the relationship between geoid anomaly and relative stress magnitudes that provide insights into local sources of stress. For example, stresses are considerably more compressive than their surroundings in some intraplate seismic zones, notably in southeastern Missouri (the area of the Reelfoot rift and the New Madrid seismic zone) and southwest Oklahoma (around the Meers fault), as well as the border between Utah, Colorado, and Wyoming. These areas are underlain by isolated zones where a pronounced negative velocity gradient observed in *S*-to-*P* receiver function studies^36^ is unusually deep (>120 km) within the subcrustal mantle lithosphere. The geologic significance of this gradient zone in the central USA remains subject to debate and may represent a mid-lithospheric discontinuity rather than the bottom of the lithosphere^37^.

Although (as noted above) thicker lithosphere is generally expected to decrease GPE, adding compressive stresses to the upper crust, there is in general not a close correlation between thickness of the thermal lithosphere^38^ (Fig. 3c) and relative stress magnitudes throughout much of the conterminous USA. Exceptions are in the Basin and Range Province, where thinner lithosphere and high heat flow^39^ are associated with normal and/or strike-slip faulting, and around northern Montana, where sharp increases in lithospheric thickness and crustal basement domain boundaries^40^ coincide closely with the transition northward to the compressive (SS/RF) stress field of western Canada. It is especially striking that the stress field is generally extensional (NF/SS) in the Great Plains considering the uniformly deep (≤150 km) lithosphere–asthenosphere boundary in the region, perhaps suggesting a shallower source of extensional stress or radial flow of asthenospheric mantle.

### Implications for sources of stress and active deformation

In parts of the northeastern USA and much of Canada, the effects of deglaciation are the primary cause of currently active deformation. However, we do not observe the patterns of faulting regime predicted by viscoelastic models of postglacial rebound. Deglaciation is expected to put the upper crust in extension in areas that were covered by the former ice sheet and to impose compression in the foreland outside the former ice sheet^8^. These models are broadly consistent with the GPS-measured strain rate field^41^, which shows dilatation under much of the region formerly covered by the Laurentide ice sheet in the northern USA and much of Canada, surrounded by a belt of contraction. The opposite pattern is observed in the stress field (Fig. 1), which shows compression (RF and SS/RF) in northeastern North America and less compressive conditions to the southwest. The profound difference between predictions and observations may be explained by stress changes induced by deglaciation superimposed on much larger ambient differential stresses. At a depth of 10 km in a SS/RF environment such as near Hudson Bay, the difference between *S*_Hmax_ and *S*_V_ is >300 MPa (assuming hydrostatic pore pressure and frictional failure equilibrium), whereas cumulative deformation at a strain rate of 3 × 10^−9^ yr^−1^ ^41^ would perturb stresses by <2 MPa after 10 ka (assuming a reasonable Young’s modulus of 60 GPa). Nevertheless, stress changes from deglaciation may affect the potential for seismicity in significant but apparently contradictory ways. Rapid reduction of overburden stress owing to removal of the ice sheet may cause large-offset but shallow earthquakes on gently dipping faults^42^ because differential stresses are relatively low near the surface and hence more readily affected by perturbations. A number of large-offset (some ≥ 5 m and up to 30 m throw) reverse faulting earthquakes on remarkably shallow faults (typically ≤ 4–8 km) in Fennoscandia^43^ and northeast Canada^44,45^ may have been triggered by this mechanism. However, lithospheric rebound may also slightly decrease the chance of deeper seismicity (e.g., >8 km) in SS/RF and RF areas by reducing horizontal stresses, which would decrease the shear stresses resolved on favorably oriented, shallowly dipping faults.

**Fig. 4:**
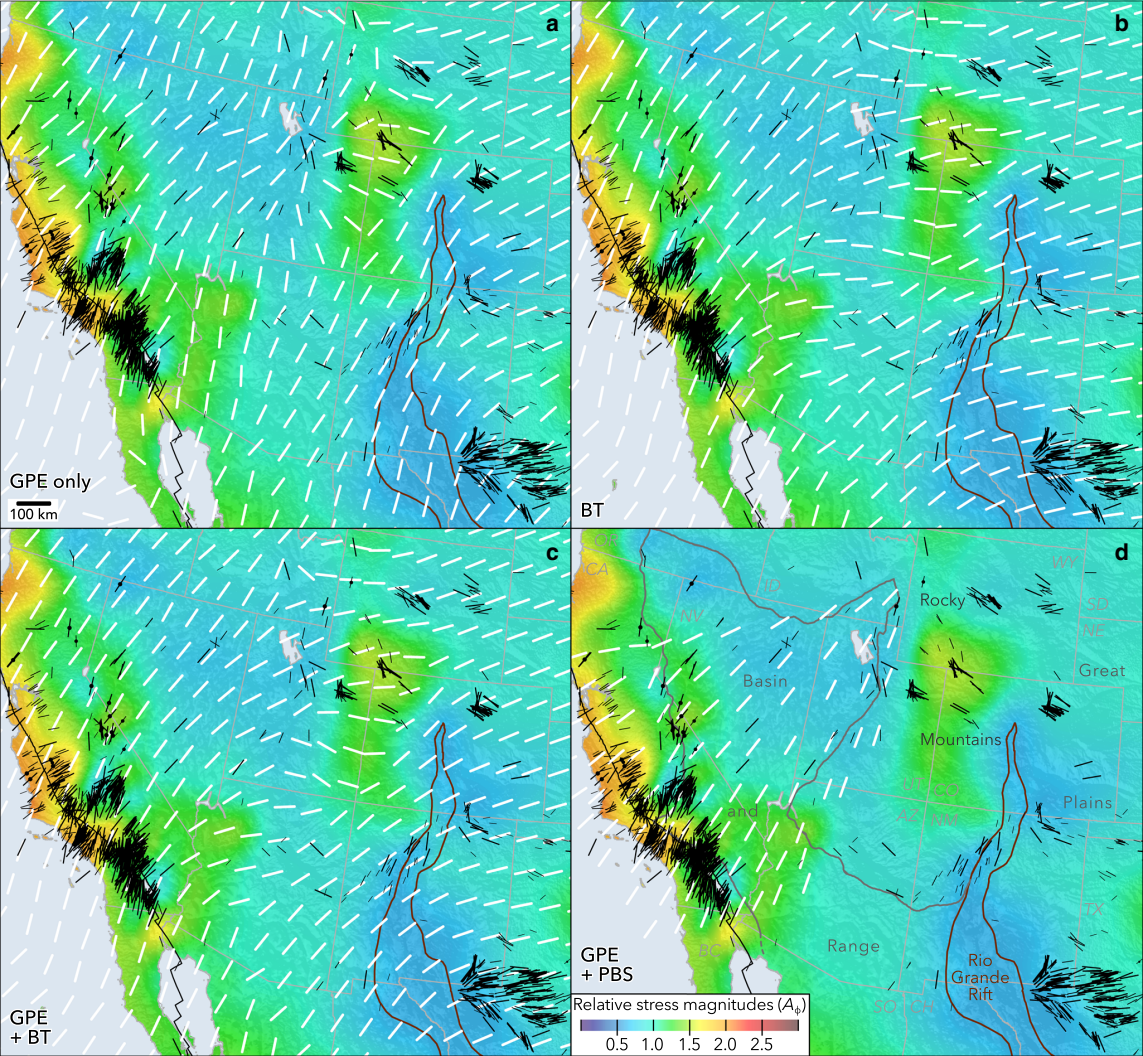
Observed (black lines and colored background) and modeled (white lines) stress fields in the southwestern USA, showing considerable spatial variability. **a**–**c** Modeled *S*_Hmax_ orientations by Ghosh et al.^4^ that account for **a** only gravitational potential energy (GPE), **b** basal tractions (BT) from modeled mantle flow, and **c** a combination of GPE and BT. **d** A model of *S*_Hmax_ orientations by Flesch et al.^47^ that considered a smaller study area incorporated only GPE and plate boundary stresses (PBS), using simpler inputs and a smaller study area.

The western USA is generally extensional, with normal and strike-slip faulting active (Fig. 4). An ongoing debate concerns the proportion of topographic elevation, stress, and deformation in this region that is attributable to dynamic mantle flow, GPE variability from lithospheric density inhomogeneities, and plate boundary interactions^1,5,21,46^. Models inferring causes of intraplate deformation indicate that the extensional stress field in this region is largely owing to elevated GPE associated with high elevations supported by buoyant lithosphere^32,47^. The detailed new stress data provide considerably better constraints for validating previously published models, and the new *S*_Hmax_ orientations provide insights for the relative importance of each proposed driving factor.

Figure 4 illustrates that *S*_Hmax_ directions vary over remarkably short wavelengths in several parts of the western USA. There are apparent ~90° changes in stress direction over 10 s of km at the margins of extensional provinces including the Rio Grande Rift and the Great Basin. Short-wavelength rotations imply shallow sources of stress at scales and magnitudes that are not accounted for in present models. The regional consistency of these short-wavelength rotations may preclude origins from transient stress perturbations associated with recent fault slip, which are typically limited to narrow zones around causative faults^48^.

As shown in Fig. 4a, published models that account only for GPE^4^ (their preferred model M2; GPE estimated from the CRUST1.0 model) are only moderately successful at matching *S*_Hmax_ orientations in the western USA. GPE-only models fit poorly in California, Arizona, and parts of Wyoming. However, these GPE-only models (Fig. 4a) are more successful than those considering basal tractions (Figs. 4b, c) in parts of the intermountain west including southeast Idaho, western Wyoming, and southwest New Mexico. Models considering GPE are in general reasonably successful at replicating to first order (although not in detail) the profound rotations of *S*_Hmax_ at the margins of the extensional provinces, including the rotation eastward to ~ENE–WSW orientations that characterize much of the central and eastern USA. This transition is best observed in the Delaware Basin (Fig. 1c), a subregion of the Permian Basin in west Texas and southeast New Mexico, where *S*_Hmax_ rotates eastward from ~N–S in southeast New Mexico near the Rio Grande Rift to ~E–W in west-central Texas^13^. Conversely, models that instead account for only basal tractions owing to modeled mantle flow^4^ (Fig. 4b) are perhaps more successful at matching *S*_Hmax_ orientations over broader scales, as is indicated by the closer match with observed *S*_Hmax_ orientations in Nevada and the western Great Plains, but less so in the intervening regions where *S*_Hmax_ rotates over shorter wavelengths. The observation that models that do not consider GPE (Fig. 4b) are the most successful in some areas, and those excluding basal tractions (Figs. 4a, d) are more successful elsewhere, suggests that the ideal weighting of these factors varies significantly across the region, and/or indicates unknown sources of stress within the lithosphere. Models that account for both GPE and basal tractions^4^ (Fig. 4c) predict patterns of *S*_Hmax_ that are quite similar to those that only consider basal tractions, further underscoring the importance of the weighting between these two factors. Finally, one model that accounts for both GPE and plate boundary tractions^47^ (Fig. 4d) is remarkably effective at replicating the observed stresses in the Great Basin and California, although it does not extend sufficiently east to cover the marked rotations of *S*_Hmax_. The similar success of models accounting for GPE plus basal tractions and GPE plus plate boundary interactions in California and Nevada suggests a degree of non-uniqueness between these factors that should be investigated by future modeling efforts.

Stream networks across the western Great Plains, east of the Rocky Mountains between southern South Dakota and west-central Texas, are undergoing active reorganization associated with recent (≤5 Ma), east-propagating uplift and tilting^49^. Although this uplift has been attributed to mantle flow^50^, the fine-scale (10’s of km) rotations of *S*_Hmax_ orientations at the western margins of the Great Plains suggest that a portion of stress variability in this region is likely owing to shallow sources of stress, likely density variations within the upper crust. The close agreement between areas of uplift and extensional stress fields (NF/SS) in the Great Plains and slightly to the west (Fig. 1), and the general relationships between the state of stress, lithospheric GPE, and topography^34^, suggest that analysis of the faulting regime may reveal other areas where elevation is currently undergoing long-term shifts.

As we have shown, quantitative characterization of the faulting regime at continental scales provides a new mechanical foundation for addressing diverse questions relating to geodynamics, geodesy, and seismic hazards. For example, quantifying the difference between forces implied by the modeled and observed stress field in various regions could help identify unaccounted for sources of stress at scales ranging from large portions of continents to 10’s of km. The profound differences between the measured strain rate and stress fields in certain areas implies that deformation observed at human time scales (such as those associated with deglaciation) may involve magnitudes and patterns dramatically different from the tectonic forces responsible for the intraplate stress field.

## Methods

### Sources and quality ratings for stress data

Figure 1 presents the first comprehensive view of the relative principal stress magnitudes (faulting regime) throughout North America, based on almost 2000 constraints (Supplementary Data 4), mostly from carefully selected focal plane mechanisms (Supplementary Data 5). The map also includes a combination of ~300 new and ~500 recently available^12,17,51,52^ *S*_Hmax_ orientations (Supplementary Data 1 and 2). Data previously published by Thompson^53,54^ from the Wind River Basin, Wyoming, are supplemented by new metadata contributed as part of this study that permit the assignment of quality ratings. Most of the new *S*_Hmax_ orientations presented as part of this study were obtained from azimuths of wellbore failure measured in subvertical wells, and others were obtained from inversions of earthquake focal mechanisms using an iterative joint inversion method^55^. Additional new indicators of horizontal stress orientations come from aligned groups of microseismic events recorded during hydraulic fracturing of horizontal wells that propagate normal to the least principal stress, which is normally *S*_hmin_^56^, and in other cases from orientations of hydraulic fractures in recently drilled horizontal wells that formed when previously drilled vertical wells in the same areas were hydraulically fractured. All measurements were assigned quality ratings according to criteria originally developed by Zoback and Zoback^6^, later adapted for the World Stress Map (WSM) project^16,18^, and updated here to incorporate the new data types. The updated quality criteria are presented in Supplementary Table 1. Higher quality ratings are assigned to measurements that sample from a larger depth range (for wellbore measurements), include greater numbers of stress indicators, and carry lower standard deviations. In addition to the new and recently available data, Fig. 1 also includes >1500 *S*_Hmax_ orientations from the WSM, which are cataloged in Supplementary Data 3. We exclude *S*_Hmax_ orientations inferred from single earthquake focal mechanism solutions owing to the considerable uncertainty in principal stress directions from individual focal plane mechanisms. In five instances noted in the Supplementary Data, we suggest modifications to WSM *S*_Hmax_ orientations owing to errors in quality assignments or indications that the measurements may be unreliable.

Relative stress magnitudes are expressed using the *A*_ϕ_ parameter following Simpson^57^. Values of *A*_ϕ_ range between 0 and 3, indicating increasingly compressive stress fields (see Fig. 1 legend). In the 50 cases where sufficient (≥20) well-constrained focal plane mechanisms were available in a localized area, we estimated *A*_ϕ_ using formal stress inversions and quantified uncertainties using bootstrap sampling. We also include ~600 previously published *A*_ϕ_ estimates, predominantly from earthquake focal mechanism inversions by Yang and Hauksson^10^. For intraplate areas lacking sufficient focal mechanism density for formalized inversions, we interpreted constraints on *A*_ϕ_ from individual mechanisms, groups of mechanisms, and/or the sense of quaternary fault offsets^58^, representing >1200 new estimates.

*S*_Hmax_ orientations and faulting regime do not typically vary significantly between wellbore measurements in sedimentary rocks and earthquake focal mechanism stress inversions from underlying crystalline rocks (as is evident in Fig. 1), although variations with depth have been observed in certain areas near active plate boundaries^59^. However, because stress magnitudes in ductile (e.g., clay-rich, organic-rich, or evaporitic) rocks can undergo viscous stress relaxation^60^, *A*_ϕ_ can vary throughout a sedimentary succession. Thus, the faulting regime mapped in Fig. 1 applies generally to the brittle (upper) crust but not necessarily to every formation within the sedimentary rocks of the upper few km. Moreover, the Gulf-normal extension (and *S*_hmin_ orientations) seen in the thick sedimentary section along the Gulf Coast may not represent those of the underlying crystalline crust as the stratigraphically deep Jurassic Louann evaporites may decouple stresses.

## Supplementary information

Supplementary Information

Description of Additional Supplementary Files

Supplementary Data 1-5

## Data Availability

All stress data presented in the figures, and the compiled catalog of >50,000 earthquake focal mechanisms used to obtain relative stress magnitudes, are available within the Supplementary Data.
