# Author Correction: Multiscale variations of the crustal stress field throughout North America

**DOI:** 10.1038/s41467-022-34915-0

**Published:** 2023-03-03

**Authors:** Jens-Erik Lund Snee, Mark D. Zoback

**Affiliations:** 1 Department of Geophysics, Stanford University, 397 Panama Mall, Mitchell Bld. 3rd Flr., Stanford, CA 94305 USA; 2 Present Address: Geosciences and Environmental Change Science Center, U.S. Geological Survey, P.O. Box 25046, MS 980, Denver, CO 25046 USA

**Keywords:** Geodynamics, Geophysics, Tectonics

Correction to: *Nature Communications* 10.1038/s41467-020-15841-5, published online 23 April 2020

Since the publication of this work, Jens-Erik Lundstern has changed his name from Jens-Erik Lund Snee. The Author has agreed that their name has to be changed back to the original. This has now been amended in the HTML and PDF versions of the article.

## Supplementary information

Updated Supplementary Information

Updated Description of Additional Supplementary Files

Updated Supplementary Data 1-5

