# Supplementary material for: Multiscale variations of the crustal stress field throughout North America
Source: Nat Commun. 2020 Apr 23;11:1951. doi: 10.1038/s41467-020-15841-5 (PMC7181828; doi:10.1038/s41467-020-15841-5)
Supplement: Supplementary file 2 — Description of Additional Supplementary Files [file 41467_2020_15841_MOESM2_ESM.pdf]

**Description of Additional Supplementary Files**

*File name:* Lundstern_Zoback_Supplementary_Information_Data.xlsx
*Description:* This supplementary data file includes Supplementary Data 1–5 as separate Excel worksheets:

- **Supplementary Data 1:** $S_{\mathrm{Hmax}}$ orientations contributed by Lundstern (formerly Lund Snee) and Zoback (this study and previously published)
- **Supplementary Data 2:** $S_{\mathrm{Hmax}}$ orientations compiled from the literature or modified from the World Stress Map
- **Supplementary Data 3:** World Stress Map data (excluding single focal mechanisms and data from Lund Snee [Lundstern] & Zoback [2016, *GRL*] and Alt & Zoback [2017, *BSSA*])
- **Supplementary Data 4:** Relative stress magnitude ($A_\phi$) control points
- **Supplementary Data 5:** Compilation of earthquake focal mechanisms
