# Supplementary material for: Author Correction: Multiscale variations of the crustal stress field throughout North America
Source: Nat Commun. 2023 Mar 3;14:1232. doi: 10.1038/s41467-022-34915-0 (PMC9984478; doi:10.1038/s41467-022-34915-0)
Supplement: Supplementary file 1 — Updated Supplementary Information [file 41467_2022_34915_MOESM1_ESM.pdf]

Supplementary Information for "Multiscale variations of the crustal stress field throughout North America"

Jens-Erik Lundstern (formerly Lund Snee)[1] & Mark D. Zoback[1]
[1]*Department of Geophysics, Stanford University, 397 Panama Mall, Mitchell Bld. 3rd Flr., Stanford, CA 94305, USA.*

Contents:

- **Supplementary Figures (1–6)**
- **Supplementary Tables**
  - **Supplementary Table 1.** Quality criteria for S_{Hmax} orientations and relative stress magnitudes (R, ϕ, or A_ϕ)
- **Supplementary Notes**
  - **Supplementary Note 1.** In situ (wellbore) measurements of S_{Hmax} orientations
  - **Supplementary Note 2.** Compilation of the focal mechanism catalog
  - **Supplementary Note 3.** Stress measurements from earthquake focal mechanism inversions
  - **Supplementary Note 4.** Interpretation of relative stress magnitudes from earthquake focal mechanisms and Quaternary fault offsets
  - **Supplementary Note 5.** Estimation of divergence angle α between S_{Hmax} orientations and absolute plate motion directions
- **Supplementary References**

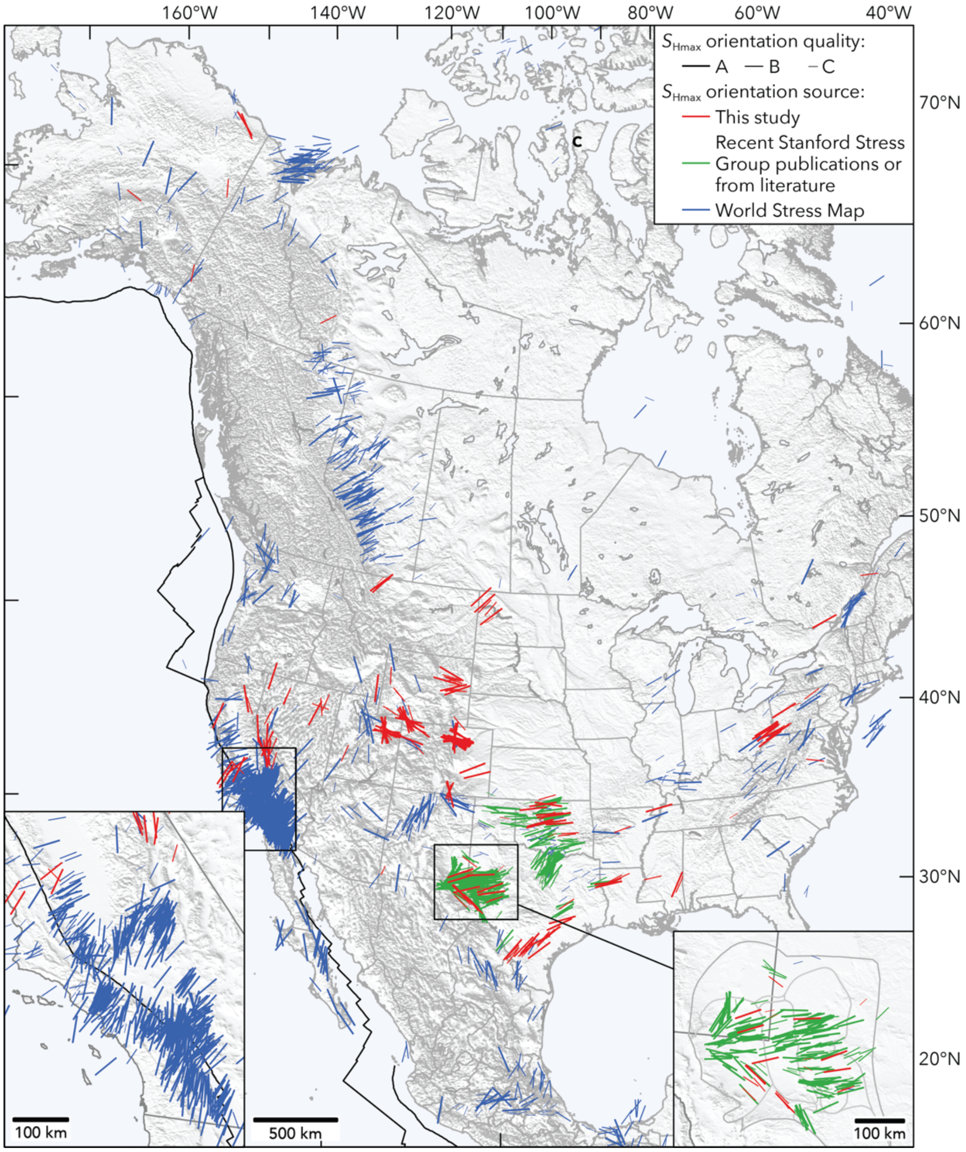

Supplementary Figure 1. Map of North America showing maximum horizontal stress (S_{Hmax}) orientations. Data that are newly presented here are in red (including those from Thompson[56,57] with new information added as part of this study), those from the World Stress Map[16] are in blue, and those compiled from recent Stanford Stress Group publications[12,13,55] and other public sources[54,101,110–115,102–109] are in green. New S_{Hmax} orientations and those compiled from published sources are tabulated in Supplementary Data 1 and 2. World Stress Map data are freely available for download at www.world-stress-map.org. Supplementary Data 3 includes WSM data from North America that are pictured in this study (i.e., excluding single focal mechanism solutions and accounting for the 5 modifications indicated in Supplementary Data 2).

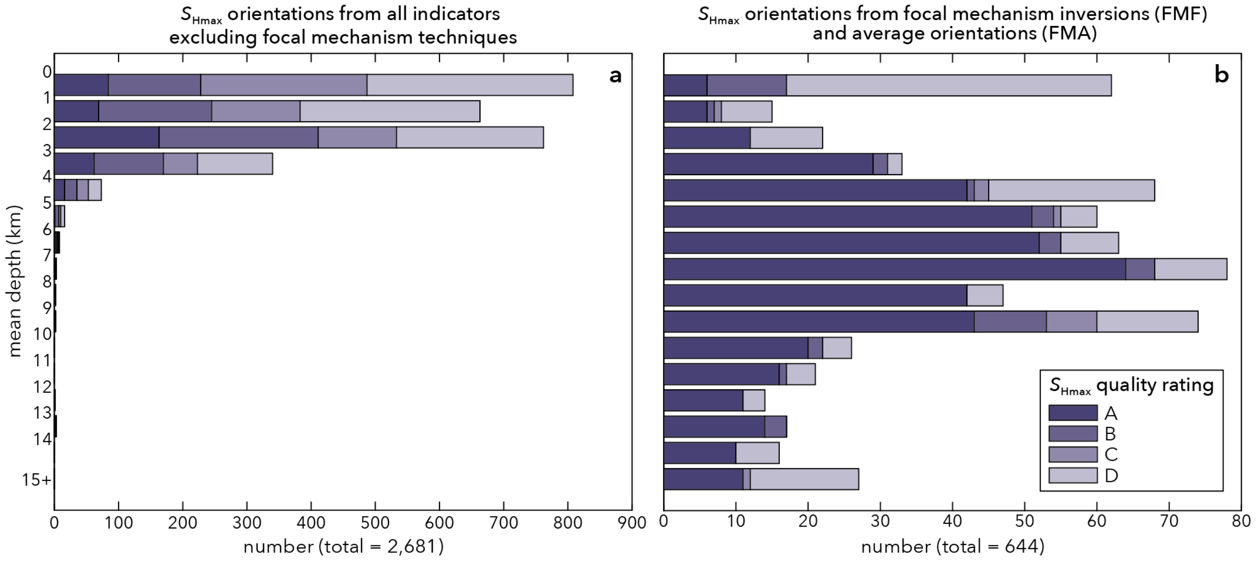

Supplementary Figure 2. Histograms illustrating the distributions of maximum horizontal stress (S_{Hmax}) orientation quality ratings with depth. **a**, Measurements from borehole techniques or aligned microseismic events from North America World Stress Map data included in Supplementary Data 3 (i.e., excluding single focal mechanism solutions), and from this and recent studies by the Stanford Stress Group[12,13,17,55]. **b**, S_{Hmax} orientations from formal focal mechanism stress inversions in North America from this study and the World Stress Map (FMF in Supplementary Data 3).

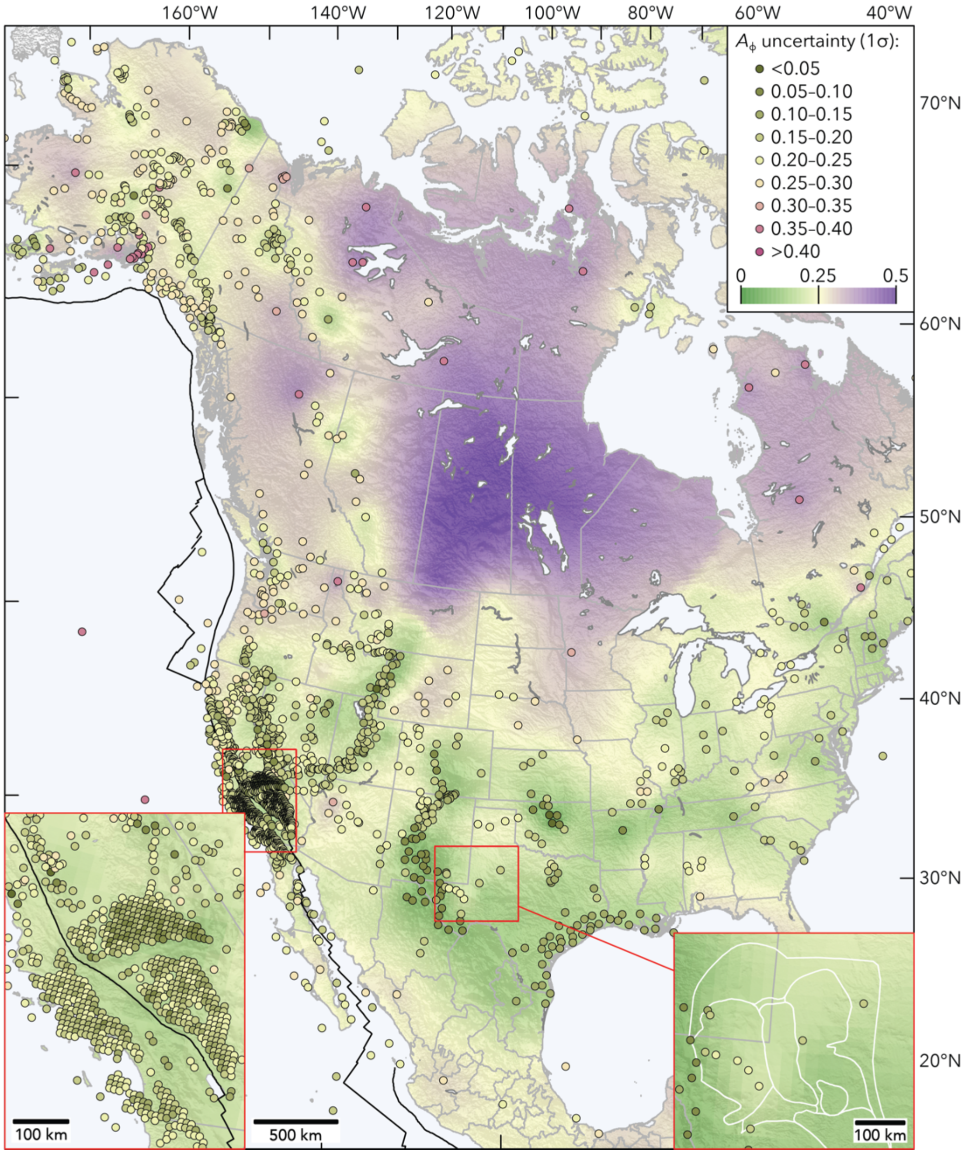

Supplementary Figure 3. Interpolated 1σ uncertainty range for A_ϕ (relative principal stress magnitude) measurements in North America. Dots are A_ϕ measurement control points colored by 1σ uncertainty, indicating A_ϕ measurement density. These data are reported in Supplementary Data 4.

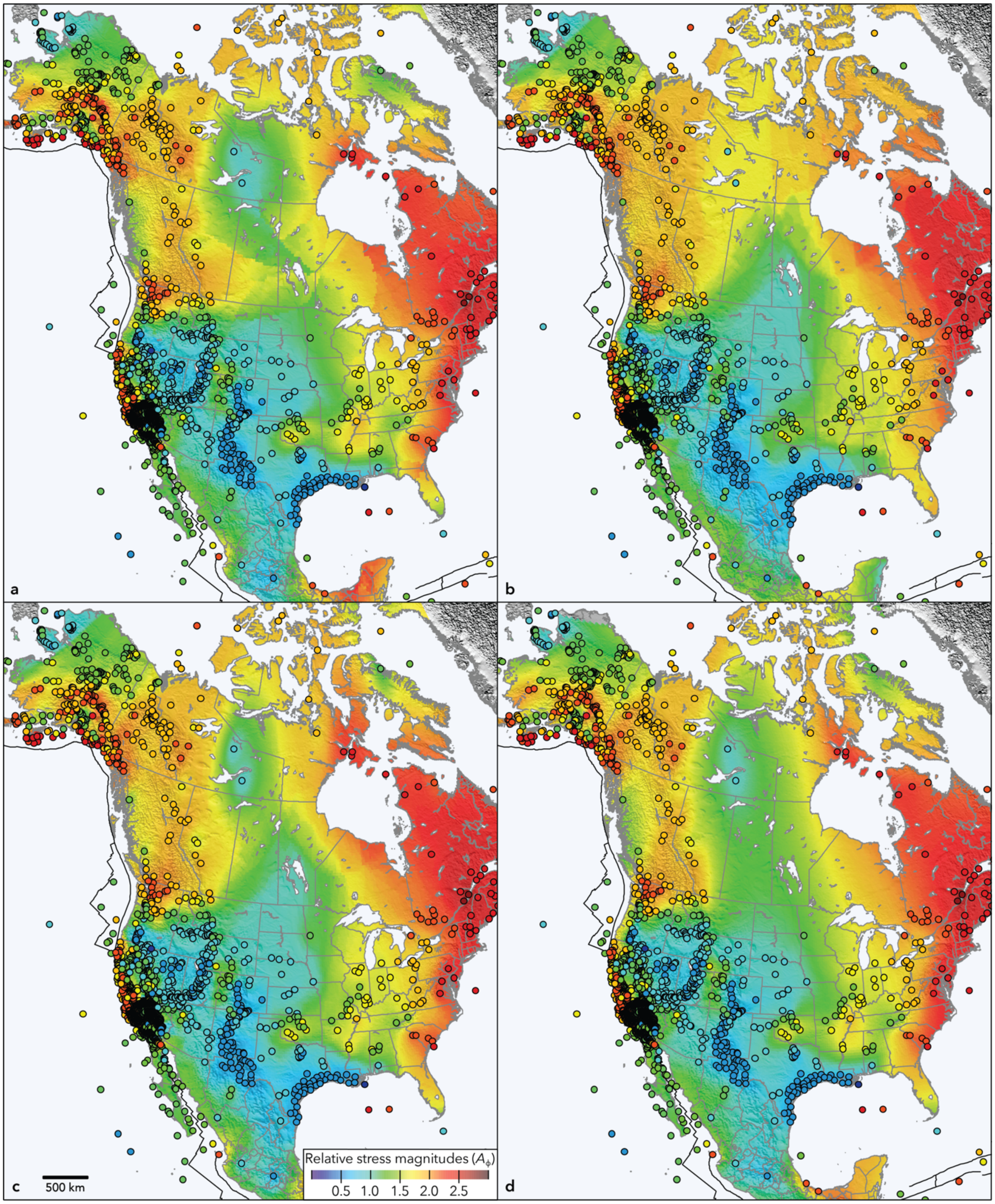

Supplementary Figure 4. Maps of A_ϕ (relative stress magnitude) in North America illustrating the effects of changing the interpolation algorithm. Unlike in the figures presented in the main body text, none of these interpolations were subsequently smoothed. Additional information is provided in the Supplementary Notes. **a**, Empirical Bayesian Kriging (the method employed for the main body figures); **b**, Ordinary Kriging; **c**, Inverse Distance Weighting; **d**, Natural Neighbor.

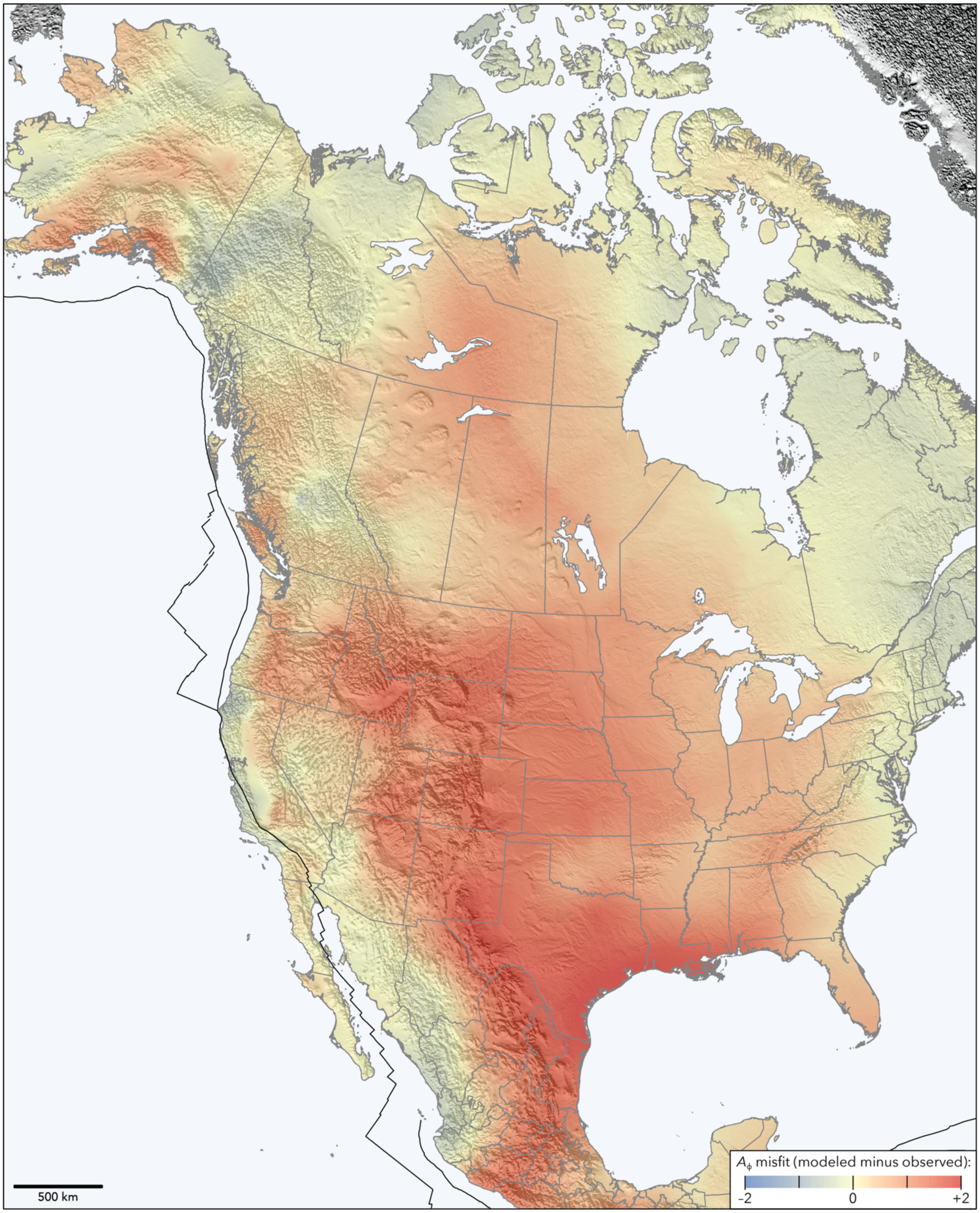

Supplementary Figure 5. Map of North America showing the difference between our A_ϕ (relative stress magnitude) values (e.g., Fig. 1) and those from the preferred global combined (GPE plus mantle flow) model (Model 4) reported by Ghosh et al.[4]. Positive values indicate areas where the predicted A_ϕ exceeds our interpolated observations. Note that uncertainties in A_ϕ that are illustrated in Supplementary Fig. 3 will affect the true misfits.

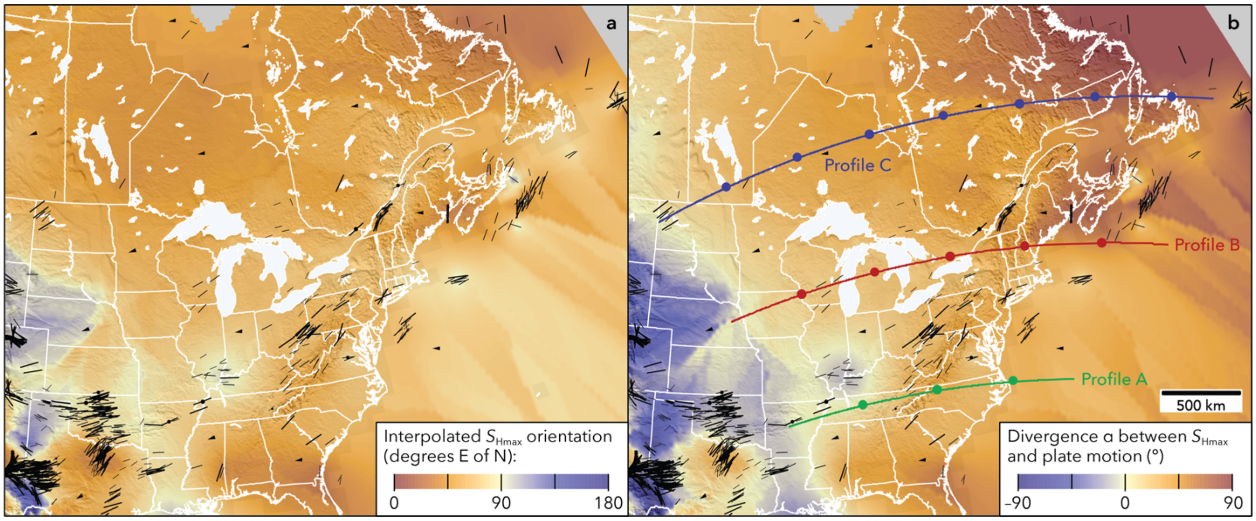

Supplementary Figure 6. Data grids used to create Fig. 2b. **a**, Maximum horizontal stress (S_{Hmax}) orientation interpolated from only A and B quality measurements. **b**, Calculated divergence angle α between S_{Hmax} and NNR-MORVEL56[19] absolute plate motion.

*STRESS INDICATOR:**		*A*	*B*	*C*
Drilling-Induced Tensile Fractures (DIF)		Ten or more distinct tensile fractures in a single well with standard deviation (sd) ≤ 12˚ and with highest and lowest observations at least 300 m apart	At least six distinct tensile fractures in a single well with sd ≤ 20˚ and with highest and lowest observations at least 100 m apart	At least four distinct tensile fractures in a single well with sd ≤ 25˚ and with highest and lowest observations at least 30 m apart
Focal Mechanism Inversions (FMF)	(Directions)	Formal inversion of ≥ 35 reasonably well-constrained focal mechanisms resulting in stress directions with sd ≤ 12º	Formal inversion of ≥ 25 reasonably well-constrained focal mechanisms resulting in stress directions with sd ≤ 20º	Formal inversion of ≥ 20 reasonably well-constrained focal mechanisms resulting in stress directions with sd ≤ 25º
	(Relative Magnitude R, ϕ, or A_ϕ)	Formal inversion of ≥ 35 reasonably well-constrained focal mechanisms resulting in ϕ with sd ≤ 0.05	Formal inversion of ≥ 25 reasonably well-constrained focal mechanisms resulting in ϕ with sd ≤ 0.1	Formal inversion of ≥ 20 reasonably well-constrained focal mechanisms resulting in ϕ with sd ≤ 0.2
Wellbore Breakouts (BO)		Ten or more distinct breakout zones in a single well (or breakouts in two or more wells in close proximity) with sd ≤ 12˚ and with highest and lowest observations at least 300 m apart	At least six distinct breakout zones in a single well with sd ≤ 20˚ and with highest and lowest observations at least 100 m apart	At least four distinct breakout zones in a single well with sd ≤ 25˚ and with highest and lowest observations at least 30 m apart
Hydraulic Fractures	Open-hole Hydraulic Fracturing Stress Orientation (HF)	Four or more hydraulic fractures in a single well (or average of hydraulic fracture orientations for two or more wells in close geographic proximity) with sd ≤ 12◦.	Three or more hydraulic fractures in a single well (or average of hydraulic fracture orientations for two or more wells in close geographic proximity) with sd ≤ 20◦.	Two or more hydraulic fracture orientations in a single well with 20◦ < sd ≤ 25◦. If a distinct orientation change with depth, the deepest measurements assumed valid.
	Hydraulic Fractures Observed in Nearby Sub-horizontal Wellbores (HFH)	Twelve or more distinct hydraulic fractures in a single well (or average of hydraulic fracture orientations for two or more wells in close geographic proximity) with sd ≤ 12º	Eight or more distinct hydraulic fractures in a single well (or average of hydraulic fracture orientations for two or more wells in close geographic proximity) with sd ≤ 20º	Six or more distinct hydraulic fractures in a single well (or average of hydraulic fracture orientations for two or more wells in close geographic proximity) with sd ≤ 25º
	Microseismic Alignments Along Hydraulic Fractures (HFM)	Twelve or more distinct linear zones associated with HF stages, with sd ≤ 12˚	Eight or more distinct linear zones associated with HF stages, with sd ≤ 20˚	Six or more distinct linear zones associated with HF stages, with sd ≤ 25˚
Shear Velocity Anisotropy from Crossed-Dipole Logs (SWA)†		Anisotropy ≥ 2% present at a consistent azimuth, with highest and lowest observations at least 300 m apart, and with sd of fast azimuth ≤ 12˚	Anisotropy ≥ 2% present at a consistent azimuth, with highest and lowest observations at least 100 m apart, and with sd of fast azimuth ≤ 20˚	Anisotropy ≥ 2% present at a consistent azimuth, with highest and lowest observations at least 30 m apart, and with sd of fast azimuth ≤ 25˚

Supplementary Table 1. Quality criteria for S_{Hmax} orientations and relative stress magnitudes (R, ϕ, or A_ϕ)

Supplementary Notes

Supplementary Note 1. In situ (wellbore) measurements of S_{Hmax} orientations

We employed established methods to obtain most *in situ* measurements of the orientation of S_{Hmax} that are newly presented in this study (Supplementary Data 1). These are supplemented by data compiled from recently published sources[12,13,16,55]. Use of such methods are explained in detail by Zoback[62]. In general, one principal stress is approximately vertical and the other two sub-horizontal because shear tractions cannot be transmitted across the interface between the solid Earth and water or air[63,64]. All S_{Hmax} orientations are shown in Supplementary Fig. 1, with symbol colors indicating data sources. Data from the World Stress Map (WSM)[16], are shown in the figures and included in Supplementary Data 3. WSM data are freely available from www.world-stress-map.org.

We applied quality ratings ranging from A (best) to D (lowest) to each S_{Hmax} orientation and to A_ϕ measurements obtained from earthquake focal mechanism inversions. Only A–C-quality measurements are considered sufficiently reliable to be plotted on stress maps (Fig. 1). Supplementary Table 1 provides the quality criteria used to apply these ratings, which are updated from previously published quality criteria[6,13,55] and newly include criteria for earthquake focal mechanism inversions. These quality criteria are very similar to those employed by the WSM[65]. The depth distributions of S_{Hmax} orientation measurements and their quality ratings throughout the continent are shown in Supplementary Fig. 2.

The bulk of the 271 reliable (A–C-quality) new S_{Hmax} orientations included in Supplementary Data 1 are from azimuths of drilling-induced tensile fractures (DIF) or borehole breakouts (BO) measured in the walls of subvertical wellbores. Such fractures, which are types of wellbore failure that often occur during drilling, develop in predictable orientations relative to the maximum and minimum horizontal principal stresses, with DIF parallel to S_{Hmax} and BO parallel to the minimum horizontal principal stress, S_{hmin} (perpendicular to S_{Hmax}). Of the new S_{Hmax} orientations, a single measurement was made from azimuths of fast shear-wave polarization measured in a subvertical well, following criteria established by Boness and Zoback[66]. This technique is based on the observation that fluid-filled fractures parallel to S_{Hmax} are typically closed, whereas those oriented perpendicular to S_{Hmax} may be slightly dilated, potentially resulting in higher shear-wave polarization velocities parallel to S_{Hmax}. An additional 64 new

S_{Hmax} orientations were obtained by averaging the azimuths of aligned groups of microseismic events produced during hydrocarbon reservoir stimulation that were thought to define propagating hydraulic fractures, as described by Warpinski et al.[67] and following techniques outlined by Lund Snee (Lundstern) and Zoback[13]. This method is based upon the expectation that hydraulic fractures open in the direction of the least principal stress, S_3, and propagate in the direction of the intermediate and maximum principal stresses, S_2 and S_1[59]. Some of these measurements were obtained from figures included in previously published papers, as referenced in Supplementary Data 1.

It is convenient that principal stress orientations and *relative* magnitudes generally do not vary significantly with depth below levels affected by topography[16,64,68,69]. Exceptions are typically due to changes to horizontal stress magnitudes due to differential fluid pressure across an impermeable fault or relaxation of the horizontal stresses due to viscous creep in ductile sedimentary layers. In addition, A_ϕ and S_{Hmax} can vary significantly within a certain distance of faults that have recently slipped[51,70]. In cases of significant variability with depth, S_{Hmax} orientations would carry high standard deviations, resulting in low ratings using the quality criteria presented in Supplementary Table 1.

Supplementary Note 2. Compilation of the focal mechanism catalog

Earthquake focal mechanisms were employed to estimate both S_{Hmax} orientations and A_ϕ, as described below. The catalog presented in Supplementary Data 1 consists of 58,709 source mechanisms from earthquakes that occurred dominantly between January 1970–March 2019, which were compiled from the sources listed below and tabulated in Supplementary Data 5. Duplicates within the merged catalog were identified by occurrence within 10 s and 0.2° latitude and longitude of one another. To ensure only crustal events, we retained only those with depths ≤ 25 km (relative to mean sea level if specified) for all catalogs except those provided by Mazzotti and Townend[71] and Singh et al.[72], and we also omitted shallower events near subduction zones that could have occurred on or below the plate interface. We applied the following additional filters to individual catalogs in order to ensure the use of only reliable mechanisms:

- Retain only $M \geq 3.0$: For International Seismological Centre (ISC) Bulletin (http://www.isc.ac.uk/iscbulletin/search/fmechanisms/);

- Use only events from Reviewed ISC Bulletin from February 1976–July 2016, and unreviewed events thereafter;
- Retain only $M \geq 3.0$: For Global Centroid Moment Tensor Project (GCMT) catalog[73,74] available via the Global CMT Catalog Search (https://www.globalcmt.org/CMTsearch.html) and the Incorporated Research Institutions for Seismology (IRIS) DMC Data Products search[75];
- Retain only $M \geq 3.5$: For U.S. Geological Survey Comprehensive Earthquake Catalog (ComCat) focal mechanisms[76];
- Retain all U.S. Geological Survey ComCat moment tensors;
- Retain only $M \geq 2.4$: For Texas Seismological Network (TexNet) mechanisms[77];
- Retain only depths ≤ 10 km in West Texas;
- Retain all Saint Louis University moment tensors[78];
- Retain only A and B quality: For Southern California earthquakes[79,80];
- Retain only $M \geq 3.0$, azimuthal gap ≤ 90º, and nearest station distance ≤ 50 km: For earthquakes from the Northern California Earthquake Data Center[81];
- Retain only misfit ≤ 0.5: For Canadian moment tensors[82–85];
- Retain all events from individual studies[71,72,81,86–90].

Supplementary Note 3. Stress measurements from earthquake focal mechanism inversions

We conducted 50 formal stress inversions using the earthquake focal mechanism catalog presented in Supplementary Data 1, yielding 46 reliable (A–C-quality) S_{Hmax} orientations (Supplementary Data 1) and 40 reliable A_ϕ (relative stress magnitude) estimates (Supplementary Data 4). These inversions were subjected to the new quality criteria set forth in Supplementary Data 1, which are informed by indications that at least 20 focal mechanisms (and often > 30), are needed to yield reliable results, particularly for A_ϕ[69,91]. Our new quality ratings additionally include a criterion for inversion uncertainties, which can be estimated using bootstrap sampling.

Formal stress inversions of earthquake focal mechanisms rely upon assumptions that the slip vector is parallel to the direction of maximum shear stress resolved on the slipping fault[92,93], that all mechanisms are reliable representations of the earthquake source geometry, that the active plane can be differentiated from the nodal plane, and that all events included as part of the same inversion occurred in a uniform stress field[94,95]. We conducted our inversions using Vavryčuk's[58] algorithm, which iteratively inverts for the active fault plane in order to maximize the accuracy

of differentiating the active and nodal planes. The 1σ error ranges and minimum and maximum values of S_{Hmax} orientations and A_ϕ were quantified using bootstrap sampling (B = 1000). The focal mechanism catalogs were filtered as described above in order to employ only reliable mechanisms. To ensure a uniform stress field for each inversion, we sought small geographic areas of events sampled for each inversion, and we avoided conducting inversions where groups of mechanisms displayed clear spatial rotations in their *P*- or *T*-axes. The conservative style of our approach to identifying sampling areas is illustrated by the relatively small number of inversions (50) that we conducted across this large region.

In our mapping, we additionally included 594 reliable A_ϕ estimates from prior focal mechanism inversions in North America made by Yang and Hauksson[10] in Southern California (their G10N30 model with 10 km squares and $\geq$ 30 mechanisms per square) and two by Quinones et al.[90] in the Fort Worth Basin, Texas. We applied our new quality criteria to these previously published inversions, excluding from our map a number of inversion results that are considered unreliable (D quality). We further excluded 3 inversions by Yang and Hauksson[10] with $A_\phi < 0.5$ (radial normal faulting) in the southern Sierra Nevada where numerous strike-slip and normal faulting earthquakes are present, as well as 1 inversion result in eastern California (in the vicinity of Owens Valley) that indicates strongly reverse faulting in spite of normal, strike-slip, and reverse faulting focal mechanisms and dominantly normal and strike-slip Quaternary fault offsets in the area. We suspect that these inversions may be unreliable due to the presence of either poorly constrained focal mechanisms or changes in the stress field within the grid boxes. The new data supersede inversion results by Mazzotti and Townend[71] in southeast Canada and the central and eastern USA because the new inversions in these areas applied the latest focal mechanisms, subject to stricter filtering criteria described above. Because of adoption of the new quality criteria, which stipulate a minimum number of focal mechanisms and maximum uncertainty bounds for inversions to be considered reliable, we did not conduct inversions in some areas for which those authors previously obtained formal A_ϕ estimates, and older inversions in those areas may be less reliable. Nevertheless, in such cases A_ϕ was interpreted informally based on the available mechanisms, using the techniques described below. In addition, we did not invert for specifically A_ϕ using TexNet mechanisms in Texas due to considerable variability in focal plane geometries in certain areas and other indications of elevated uncertainty. However,

we did formally invert for only S_{Hmax} orientations using the TexNet mechanisms based on evidence mentioned above that S_{Hmax} is less sensitive to nodal plane uncertainties[91].

Finally, because multiple plate-bounding fault zones cut North America, we note that estimates of faulting regime from earthquake focal mechanism stress inversions are unreliable if they include events that occurred on faults with anomalously low coefficients of friction. On such faults, the sense of slip (e.g., strike-slip) may differ from the faulting regime (e.g., reverse faulting), as is the case in some areas near the San Andreas fault zone[96]. Perhaps related to this effect, as well as the very high rate of seismicity in the area, Abolfathian et al.[97] have shown appreciable stress changes with depth near major faults associated with the plate boundary in Southern California. For these reasons, we do not include events that occurred within 10 km of plate bounding faults or major, potentially weak subsidiary structures near plate boundary zones. We also exclude previously published A_ϕ inversion results that include such earthquakes.

Supplementary Note 4. Interpretation of relative stress magnitudes from earthquake focal mechanisms and Quaternary fault offsets

Of the 1303 new estimates of A_ϕ (relative stress magnitudes), 1263 are based on the slip sense of Quaternary fault offsets, observations of fault slip sense from microseismic events and focal mechanisms monitored during hydraulic fracturing operations, or interpretations from individual earthquake focal plane mechanisms or groups of mechanisms (Supplementary Data 4). Earthquake focal mechanisms and paleoseismic indicators provide constraints on A_ϕ even in cases where there are too few indicators to conduct a formal inversion. Observation of a specific sense of fault slip (normal, strike-slip, reverse, or oblique) provides permissible and impermissible ranges of A_ϕ; multiple observations in an area allow for interpretation of increasingly well-constrained permissible A_ϕ ranges. For example, nearby occurrence of both normal and strike-slip faulting (and/or oblique normal/strike-slip events) indicates a faulting regime between normal faulting ($A_\phi = 0.5$) and strike-slip faulting ($A_\phi = 1.5$), bounding A_ϕ between 0.5–1.5. Similarly, the presence of a single normal faulting mechanism indicates a faulting regime between normal/strike-slip faulting ($A_\phi \leq \sim1.25$) and the extremely rare condition of radial normal faulting ($A_\phi = 0$). The uncertainty bounds can be narrowed considerably in cases where multiple slip sense observations are available. For example, numerous events distributed

roughly equally between normal to strike-slip faulting sense (or numerous oblique normal/strike-slip events) suggest that A_ϕ is likely near 1.0.

Based on this framework, we interpreted A_ϕ and its uncertainty using the information provided by earthquake focal mechanisms and Quaternary fault offsets, with interpretations informed by formal focal mechanism stress inversions. We assumed normal uncertainty distributions for each A_ϕ measurement, for which we interpret the mean (μ, presented in Fig. 1), standard deviation (1σ, illustrated in Supplementary Fig. 3), and minimum and maximum truncation bounds ($t_{\min}$ and $t_{\max}$) for the distribution.

Data for recent fault slip sense were drawn from the U.S. Geological Survey Quaternary Fault and Fold Database[60], as well as evidence for reverse–strike-slip faulting in the paleoseismic record of the Meers Fault, southwest Oklahoma[98,99], and extensional growth faulting along the Gulf Coast margin[64]. Focal mechanisms were compiled as described above. In general, focal mechanisms were considered more reliable than Quaternary offsets due to the lower precision of the fault offset record (e.g., slip vectors are not typically recorded for Quaternary offsets, limiting the potential to recognize oblique slip) and a potential bias against sampling strike-slip faults due to greater challenges identifying offsets without significant vertical components.

A_ϕ was interpolated using the Empirical Bayesian Kriging (EBK) algorithm supplied in the ESRI ArcGIS v.10.6.1 software program (Geostatistical Analyst toolbox), using a power variogram model and standard circular search neighborhood with a minimum of 10 neighbors and a maximum of 15 neighbors. The interpolation was made on the mean A_ϕ value (μ) given in Supplementary Data 4. To illustrate the uncertainty range (Supplementary Fig. 3), the 1σ measurement-point uncertainties given in Supplementary Data 1 were interpolated using the same algorithm. Both interpolations were smoothed slightly using a low-pass filter with a 3×3 window. Supplementary Fig. 4 compares the interpolation using EBK to other options such as Natural Neighbor, Inverse Distance Weighted (IDW), and Ordinary Kriging. The latter three algorithms are those provided by ArcGIS v.10.6.1 (Spatial Analyst toolbox), and default parameters were employed for each. Unlike in the figures presented in the main body text, the A_ϕ interpolations in this comparison figure were not subsequently smoothed. The difference between our interpolated mean A_ϕ values and the A_ϕ values predicted from the preferred global combined (GPE plus mantle flow) model (Model 4) reported by Ghosh et al.[4]. are shown in Supplementary Fig. 5.

Supplementary Note 5. Estimation of divergence angle α between S_{Hmax} orientations and absolute plate motion directions

S_{Hmax} orientations were gridded using EBK with the same parameters as in the interpolation of A_ϕ (see above). To reduce noise, only higher (A and B) quality S_{Hmax} orientations were used for the interpolation. The resulting grid is presented in Supplementary Fig. 6a. The S_{Hmax} orientation raster was subtracted from a grid of orientations of NNR-MORVEL56 absolute plate motions[19] using the ArcGIS Raster Calculator. The resulting grid of divergence angles (Supplementary Fig. 6b) was smoothed slightly using a 3 × 3 low-pass filter. Divergence angles were extracted from this grid along three 2º-wide swath profiles (Fig. 2) that were constructed approximately parallel to plate motion using TopoToolbox v.2.3[100] in MATLAB. Divergence angles were sampled every 0.1º along and across the swath profiles. The negative mean across-profile value for each point along the profile is shown in Fig. 2b.

Supplementary References

1. Molnar, P., England, P. C. & Jones, C. H. Mantle dynamics, isostasy, and the support of high terrain. *J. Geophys. Res. Solid Earth* **120**, 1932–1957 (2015).
2. Levandowski, W., Zellman, M. & Briggs, R. W. Gravitational body forces focus North American intraplate earthquakes. *Nat. Commun.* **8**, 14314 (2017).
3. Eakin, C. M. & Lithgow-Bertelloni, C. An Overview of Dynamic Topography: The Influence of Mantle Circulation on Surface Topography and Landscape. *Mt. Clim. Biodivers.* 37 (2018).
4. Ghosh, A., Holt, W. E. & Bahadori, A. Role of Large-Scale Tectonic Forces in Intraplate Earthquakes of Central and Eastern North America. *Geochemistry, Geophys. Geosystems* **20**, 2134–2156 (2019).
5. Zhou, Q. & Liu, L. Topographic evolution of the western United States since the early Miocene. *Earth Planet. Sci. Lett.* **514**, 1–12 (2019).
6. Zoback, M. Lou & Zoback, M. D. Tectonic Stress Field of the Continental United States. in *Geological Society of America Memoirs* **172**, 523–540 (1989).
7. Stein, S., Cloetingh, S. A. P. L., Sleep, N. H. & Wortel, R. Passive Margin Earthquakes, Stresses and Rheology. in *Earthquakes at North-Atlantic Passive Margins: Neotectonics and Postglacial Rebound* **266**, 231–259 (Springer Netherlands, 1989).
8. Stewart, I. S., Sauber, J. & Rose, J. Glacio-seismotectonics: Ice sheets, crustal deformation and seismicity. *Quat. Sci. Rev.* **19**, 1367–1389 (2000).
9. Ruppert, N. A. Stress Map for Alaska From Earthquake Focal Mechanisms. 351–367 (2008).
10. Yang, W. & Hauksson, E. The tectonic crustal stress field and style of faulting along the Pacific North America plate boundary in southern California. *Geophys. J. Int.* **194**, 100–117 (2013).
11. Hurd, O. & Zoback, M. D. Intraplate earthquakes, regional stress and fault mechanics in the Central and Eastern U.S. and Southeastern Canada. *Tectonophysics* **581**, 182–192 (2012).
12. Lund Snee, J.-E. & Zoback, M. D. State of stress in Texas: Implications for induced seismicity. *Geophys. Res. Lett.* **43**, 10,208-10,214 (2016).

13. Lund Snee, J.-E. & Zoback, M. D. State of stress in the Permian Basin, Texas and New Mexico: Implications for induced seismicity. *Lead. Edge* 810–819 (2018). doi:10.1190/tle37020127.1
14. Levandowski, W., Herrmann, R. B., Briggs, R. W., Boyd, O. S. & Gold, R. D. An updated stress map of the continental United States reveals heterogeneous intraplate stress. *Nat. Geosci.* **11**, 433–437 (2018).
15. Townend, J. & Zoback, M. D. Regional tectonic stress near the San Andreas fault in central and southern California. *Geophys. Res. Lett.* **31**, 1–5 (2004).
16. Heidbach, O. *et al.* The World Stress Map database release 2016: Crustal stress pattern across scales. *Tectonophysics* **744**, 484–498 (2018).
17. Hennings, P. H. *et al.* Injection-Induced Seismicity and Fault-Slip Potential in the Fort Worth Basin, Texas. *Bull. Seismol. Soc. Am.* (2019). doi:10.1785/0120190017
18. Simpson, R. W. Quantifying Anderson's fault types. *J. Geophys. Res.* **102**, 17909–17919 (1997).
19. Argus, D. F., Gordon, R. G. & Demets, C. Geologically current motion of 56 plates relative to the no-net-rotation reference frame. *Geochemistry, Geophys. Geosystems* **12**, 1–13 (2011).
20. Zoback, M. Lou. First- and second-order patterns of stress in the lithosphere: The World Stress Map Project. *J. Geophys. Res.* **97**, 11703–11728 (1992).
21. Artyushkov, E. V. Stresses in the lithosphere caused by crustal thickness inhomogeneities. *J. Geophys. Res.* **78**, 7675–7708 (1973).
22. Bott, M. H. P. Ridge push and associated plate interior stress in normal and hot spot regions. *Tectonophysics* **200**, 17–32 (1991).
23. Ghosh, A., Holt, W. E. & Flesch, L. M. Contribution of gravitational potential energy differences to the global stress field. *Geophys. J. Int.* **179**, 787–812 (2009).
24. Sonder, L. J. Effects of density contrasts on the orientation of stresses in the lithosphere: Relation to principal stress directions in the Transverse Ranges, California. *Tectonics* **9**, 761–771 (1990).
25. Zoback, M. Lou & Mooney, W. D. Lithospheric Buoyancy and Continental Intraplate Stresses. *Int. Geol. Rev.* **45**, 95–118 (2003).
26. Poudjom Djomani, Y. H., O'Reilly, S. Y., Griffin, W. L. & Morgan, P. The density

structure of subcontinental lithosphere through time. *Earth Planet. Sci. Lett.* **184**, 605–621 (2001).

27. Mitrovica, J. X. & Peltier, W. R. Pleistocene deglaciation and the global gravity field. *J. Geophys. Res.* **94**, 13651–13671 (1989).
28. Forsyth, D. W. & Uyeda, S. On the Relative Importance of the Driving Forces of Plate Motion. *Geophys. J. Int.* **43**, 163–200 (1975).
29. Evans, K. F. Appalachian Stress Study: 3. Regional scale stress variations and their relation to structure and contemporary tectonics. *J. Geophys. Res.* **94**, 17619 (1989).
30. Kucks, R. P. Bouguer gravity anomaly data grid for the conterminous US. (1999).
31. Mooney, W. D. & Kaban, M. K. The North American upper mantle: Density, composition, and evolution. *J. Geophys. Res. Solid Earth* **115**, 1–24 (2010).
32. McGarr, A. F. Analysis of states of stress between provinces of constant stress. *J. Geophys. Res.* **87**, 9279 (1982).
33. Humphreys, E. D. & Coblentz, D. D. North American Dynamics and Western U . S . *Rev. Geophys.* **45**, 1–30 (2007).
34. Shen, W. & Ritzwoller, M. H. Crustal and uppermost mantle structure beneath the United States. *J. Geophys. Res. Solid Earth* **121**, 4306–4342 (2016).
35. Chase, C. G., Libarkin, J. A. & Sussman, A. J. Colorado Plateau: Geoid and Means of Isostatic Support. *Int. Geol. Rev.* **44**, 575–587 (2002).
36. Richards, M. A. & Hager, B. H. Geoid anomalies in a dynamic Earth. *J. Geophys. Res. Solid Earth* **89**, 5987–6002 (1984).
37. Kind, R., Yuan, X. & Kumar, P. Seismic receiver functions and the lithosphere-asthenosphere boundary. *Tectonophysics* **536–537**, 25–43 (2012).
38. Fischer, K. M., Ford, H. A., Abt, D. L. & Rychert, C. A. The Lithosphere-Asthenosphere Boundary. *Annu. Rev. Earth Planet. Sci.* **38**, 551–575 (2010).
39. Artemieva, I. M. Global 1° × 1° thermal model TC1 for the continental lithosphere: Implications for lithosphere secular evolution. *Tectonophysics* **416**, 245–277 (2006).
40. Blackwell, D. D. *et al. SMU Geothermal Laboratory Heat Flow Map of the Conterminous United States*. (2011).
41. Lund, K. *et al.* Basement Domain Map of the Conterminous United States and Alaska. *U.S. Geol. Surv. Data Ser. 898* 41 (2015). doi:10.3133/ds898

42. Pindell, J. L. & Kennan, L. Tectonic evolution of the Gulf of Mexico, Caribbean and northern South America in the mantle reference frame: an update. *Geol. Soc. London, Spec. Publ.* **328**, 1.1-55 (2009).

43. Pavlis, N. K., Holmes, S. A., Kenyon, S. C. & Factor, J. K. The development and evaluation of the Earth Gravitational Model 2008 (EGM2008). *J. Geophys. Res. Solid Earth* **118**, 2633 (2013).

44. Kreemer, C., Hammond, W. C. & Blewitt, G. A Robust Estimation of the 3-D Intraplate Deformation of the North American Plate From GPS. *J. Geophys. Res. Solid Earth* **123**, 4388–4412 (2018).

45. Muir Wood, R. Extraordinary Deglaciation Reverse Faulting in Northern Fennoscandia. in *Earthquakes at North-Atlantic Passive Margins: Neotectonics and Postglacial Rebound* 141–173 (Springer Netherlands, 1989). doi:10.1007/978-94-009-2311-9_10

46. Malehmir, A. *et al.* Post-glacial reactivation of the Bollnäs fault, central Sweden - A multidisciplinary geophysical investigation. *Solid Earth* **7**, 509–527 (2016).

47. Stein, S., Sleep, N. H., Geller, R. J., Wang, S. & Kroeger, G. C. Earthquakes along the passive margin of eastern Canada. *Geophys. Res. Lett.* **6**, 537–540 (1979).

48. Adams, J., Wetmiller, R. J., Hasegawa, H. S. & Drysdale, J. The first surface faulting from a historical intraplate earthquake in North America. *Nature* **352**, 617–619 (1991).

49. Liu, Z. & Bird, P. North America plate is driven westward by lower mantle flow. *Geophys. Res. Lett.* **29**, 17-1-17–4 (2002).

50. Flesch, L. M., Holt, W. E., Haines, A. J. & Shen-Tu, B. Dynamics of the Pacific-North American Plate Boundary in the Western United States. *Science (80-.).* **287**, 834–836 (2000).

51. Castillo, D. A. & Zoback, M. D. Systematic variations in stress state in the southern San Joaquin Valley: inferences based on well-bore data and contemporary seismicity. *Am. Assoc. Pet. Geol. Bull.* **78**, 1257–1275 (1994).

52. Willett, S. D., McCoy, S. W. & Beeson, H. W. Transience of the North American High Plains landscape and its impact on surface water. *Nature* **561**, 528–532 (2018).

53. Moucha, R. *et al.* Deep mantle forces and the uplift of the Colorado Plateau. *Geophys. Res. Lett.* **36**, 1–6 (2009).

54. Forand, D., Heesakkers, V., Schwartz, K., Tx, H. & States, U. Constraints on natural

fracture and in-situ stress trends of unconventional reservoirs in the Permian Basin , USA. *Unconv. Resour. Technol. Conf.* (2017). doi:10.15530/-urtec-2017-2669208

55. Alt, R. C. & Zoback, M. D. In situ stress and active faulting in Oklahoma. *Bull. Seismol. Soc. Am.* **107**, 216–228 (2017).

56. Thompson, R. C. Two-stage development of the Wind River Basin, Wyoming: Laramide shortening followed by post-Laramide regional extension, localized backsliding, and arch collapse. (Colorado State University, 2010).

57. Thompson, R. C. Post-Laramide, Collapse-Related Fracturing and Associated Production; Wind River Basin, Wyoming. *Rocky Mt. Geol.* **52**, 27–46 (2015).

58. Vavryčuk, V. Iterative joint inversion for stress and fault orientations from focal mechanisms. *Geophys. J. Int.* **199**, 69–77 (2014).

59. Hubbert, M. K. & Willis, D. G. Mechanics of hydraulic fracturing. *AAPG Mem.* **18**, 239–257 (1972).

60. Crone, A. J. & Wheeler, R. L. Data for Quaternary faults, liquefaction features, and possible tectonic features in the Central and Eastern United States, east of the Rocky Mountain front. *U.S. Geol. Surv. Open-File Rep. 00-260* 332 (2000).

61. Sone, H. & Zoback, M. D. Time-dependent deformation of shale gas reservoir rocks and its long-term effect on the in situ state of stress. *Int. J. Rock Mech. Min. Sci.* **69**, 120–132 (2014).

62. Zoback, M. D. *Reservoir Geomechanics*. (Cambridge University Press, 2010). doi:doi:10.1017/CBO9780511586477

63. Anderson, E. M. *The dynamics of faulting and dyke formation with applications to Britain*. (Hafner Pub. Co., 1951).

64. Zoback, M. Lou & Zoback, M. D. State of stress in the conterminous United States. *J. Geophys. Res.* **85**, 6113–6156 (1980).

65. Heidbach, O., Rajabi, M., Reiter, K. & Ziegler, M. O. The 2016 release of the World Stress Map. (2016). doi:10.5880/WSM.2016.002

66. Boness, N. L. & Zoback, M. D. Stress-induced seismic velocity anisotropy and physical properties in the SAFOD Pilot Hole in Parkfield, CA. *Geophys. Res. Lett.* **31**, 15–18 (2004).

67. Warpinski, N., Branagan, P. T., Peterson, R. E., Wolhart, S. L. & Uhl, J. E. Mapping

Hydraulic Fracture Growth and Geometry Using Microseismic Events Detected by a Wireline Retrievable Accelerometer Array. *SPE Gas Technol. Symp.* 335–346 (1998). doi:10.2118/40014-MS

68. Brudy, M., Zoback, M. D., Fuchs, K., Rummel, F. & Baumgärtner, J. Estimation of the complete stress tensor to 8 km depth in the KTB scientific drill holes: Implications for crustal strength. *J. Geophys. Res. Solid Earth* **102**, 18453–18475 (1997).
69. Walsh, F. R. I. & Zoback, M. D. Probabilistic assessment of potential fault slip related to injection-induced earthquakes: Application to north-central Oklahoma, USA. *Geology* **44**, 991–994 (2016).
70. Hickman, S. H. *et al.* Stress and permeability heterogeneity within the Dixie Valley geothermal reservoir: Recent results from well 82-5. *Proc. 25th Work. Geotherm. Reserv. Eng.* 256–265 (2000).
71. Mazzotti, S. & Townend, J. State of stress in central and eastern North American seismic zones. *Lithosphere* **2**, 76–83 (2010).
72. Singh, S. K., Pacheco, J. F., Pérez-Campos, X., Ordaz, M. & Reinoso, E. The 6 September 1997 (Mw4.5) Coatzacoalcos-Minatitlán, Veracruz, Mexico earthquake: implications for tectonics and seismic hazard of the region. *Geofísica Int.* **54**, 289–298 (2015).
73. Dziewoński, A. M., Chou, T.-A. & Woodhouse, J. H. Determination of earthquake source parameters from waveform data for studies of global and regional seismicity. *J. Geophys. Res. Solid Earth* **86**, 2825–2852 (1981).
74. Ekström, G., Nettles, M. & Dziewoński, A. M. The global CMT project 2004-2010: Centroid-moment tensors for 13,017 earthquakes. *Phys. Earth Planet. Inter.* **200–201**, 1–9 (2012).
75. Trabant, C. ~M. *et al.* Data Products at the IRIS DMC: Stepping Stones for Research and Other Applications. *Seismol. Res. Lett.* **83**, 846–854 (2012).
76. U.S. Geological Survey Earthquake Hazards Program. Advanced National Seismic System (ANSS) Comprehensive Catalog of Earthquake Events and Products. (2017). Available at: https://doi.org/10.5066/F7MS3QZH.
77. Savvaidis, A., Young, B., Huang, G. D. & Lomax, A. TexNet: A Statewide Seismological Network in Texas. *Seismol. Res. Lett.* **90**, (2019).
78. Herrmann, R. B., Benz, H. M. & Ammon, C. J. Monitoring the earthquake source process

in North America. *Bull. Seismol. Soc. Am.* **101**, 2609–2625 (2011).

79. Hauksson, E., Yang, W. & Shearer, P. M. Waveform relocated earthquake catalog for Southern California (1981 to June 2011). *Bull. Seismol. Soc. Am.* **102**, 2239–2244 (2012).

80. Yang, W., Hauksson, E. & Shearer, P. M. Computing a large refined catalog of focal mechanisms for southern California (1981-2010): Temporal stability of the style of faulting. *Bull. Seismol. Soc. Am.* **102**, 1179–1194 (2012).

81. Braunmiller, J. & Nábělek, J. Seismotectonics of the Explorer region. *J. Geophys. Res. Solid Earth* **107**, ETG 1-1-ETG 1-25 (2002).

82. Kao, H., Jian, P.-R., Ma, K.-F., Huang, B.-S. & Liu, C.-C. Moment-tensor inversion for offshore earthquakes east of Taiwan and their implications to regional collision. *Geophys. Res. Lett.* **25**, 3619–3622 (1998).

83. Kao, H. & Jian, P.-R. Source Parameters of Regional Earthquakes in Taiwan: July 1995-December 1996. *Terr. Atmos. Ocean. Sci.* **10**, 585 (1999).

84. Ristau, J., Rogers, G. C. & Cassidy, J. F. Moment magnitude-local magnitude calibration for earthquakes off Canada's West Coast. *Bull. Seismol. Soc. Am.* **93**, 2296–2300 (2003).

85. Kao, H. *et al.* Regional Centroid-Moment-Tensor Analysis for Earthquakes in Canada and Adjacent Regions: An Update. *Seismol. Res. Lett.* **83**, 505–515 (2012).

86. Ichinose, G. A., Anderson, J. G., Smith, K. D. & Zeng, Y. Source parameters of eastern California and western Nevada earthquakes from regional moment tensor inversion. *Bull. Seismol. Soc. Am.* **93**, 61–84 (2003).

87. Ristau, J., Rogers, G. C. & Cassidy, J. F. Stress in western Canada from regional moment tensor analysis. *Can. J. Earth Sci.* **44**, 127–148 (2007).

88. Eaton, D. W. & Mahani, A. B. Focal Mechanisms of Some Inferred Induced Earthquakes in Alberta, Canada. *Seismol. Res. Lett.* **86**, 1078–1085 (2015).

89. Scales, M. M. *et al.* A Decade of Induced Slip on the Causative Fault of the 2015 Mw 4.0 Venus Earthquake, Northeast Johnson County, Texas. *J. Geophys. Res. Solid Earth* **122**, 7879–7894 (2017).

90. Quinones, L. A., DeShon, H. R., Magnani, M. B. & Frohlich, C. Stress orientations in the Fort Worth Basin, Texas, determined from earthquake focal mechanisms. *Bull. Seismol. Soc. Am.* **108**, 1124–1132 (2018).

91. Martínez-Garzón, P., Ben-Zion, Y., Abolfathian, N., Kwiatek, G. & Bohnhoff, M. A

refined methodology for stress inversions of earthquake focal mechanisms. *J. Geophys. Res. Solid Earth* 1–22 (2016). doi:10.1002/2016JB013493

92. Wallace, R. E. Geometry of shearing stress and relation to faulting. *J. Geol.* **59**, 118–130 (1951).
93. Bott, M. H. P. The mechanics of oblique slip faulting. *Geol. Mag.* **106**, 109–117 (1959).
94. Michael, A. J. Determination of stress from slip data: Faults and folds. *J. Geophys. Res.* **89**, 11517 (1984).
95. Michael, A. J. Use of focal mechanisms to determine stress: A control study. *J. Geophys. Res.* **92**, 357–368 (1987).
96. Zoback, M. D. *et al.* New evidence on the state of stress of the San Andreas fault system. *Science (80-.).* **238**, 1105–1111 (1987).
97. Abolfathian, N., Martínez-Garzón, P. & Ben-Zion, Y. Spatiotemporal Variations of Stress and Strain Parameters in the San Jacinto Fault Zone. *Pure Appl. Geophys.* **176**, 1145–1168 (2018).
98. Madole, R. F. Stratigraphic evidence of Holocene faulting in the mid-continent: The Meers fault, southwestern Oklahoma. *Geol. Soc. Am. Bull.* **100**, 392–401 (1988).
99. Crone, A. J. & Luza, K. V. Style and timing of Holocene surface faulting on the Meers fault, southwestern Oklahoma. *Bull. Geol. Soc. Am.* **102**, 1–17 (1990).
100. Schwanghart, W. & Scherler, D. Short Communication: TopoToolbox 2 – MATLAB-based software for topographic analysis and modeling in Earth surface sciences. *Earth Surf. Dyn.* **2**, 1–7 (2014).
101. Davatzes, N. C. & Hickman, S. H. Preliminary analysis of stress in the newberry EGS Well NWG 55-29. *Trans. - Geotherm. Resour. Counc.* **35 1**, 323–332 (2011).
102. Kessler, J. A. *et al.* Geology and in situ stress of the MH-2 borehole, Idaho, USA: Insights into western Snake River Plain structure from geothermal exploration drilling. *Lithosphere* **9**, 476–498 (2017).
103. Alalli, A. A. & Zoback, M. D. Microseismic evidence for horizontal hydraulic fractures in the Marcellus Shale, southeastern West Virginia. *Lead. Edge* **37**, 356–361 (2018).
104. Sone, H. Mechanical properties of shale gas reservoir rocks and its relation to the in-situ stress variation observed in shale gas reservoirs. (Stanford University, 2012).
105. Xu, S. & Zoback, M. D. Analysis of stress variations with depth in the Permian Basin

Spraberry / Dean / Wolfcamp Shale. *Am. Rock Mech. Assoc.* **15–189**, (2015).

106. Robertson-Tait, A., Lutz, S. J., Sheridan, J. & Morris, C. L. Selection of an interval for massive hydraulic stimulation in well DP 23-1 Desert Peak East EGS Project, Nevada. *Proc. Twenty-Ninth Work. Geotherm. Reserv. Eng. Stanford Univ.* **29**, 216–221 (2004).
107. Heller, R. J. Multiscale investigation of fluid transport in gas shales. (Stanford University, 2013).
108. Sturm, S. D. & Gomez, E. Role of natural fracturing in production from the Bakken Formation, Williston basin North Dakota. in *AAPG Annual Convention and Exhibition, Denver, Colorado* **50199**, #50199 (2009).
109. Fan, Z., Eichhubl, P. & Gale, J. F. W. Geomechanical analysis of fluid injection and seismic fault slip for the Mw 4.8 Timpson, Texas, earthquake sequence. *J. Geophys. Res. Solid Earth* **121**, 2798–2812 (2016).
110. Vermylen, J. Geomechanical studies of the Barnett shale, Texas, USA. (Stanford University, 2011).
111. Jolie, E., Moeck, I. & Faulds, J. E. Quantitative structural-geological exploration of fault-controlled geothermal systems-A case study from the Basin-and-Range Province, Nevada (USA). *Geothermics* **54**, 54–67 (2015).
112. Raterman, K. T. *et al.* Sampling a Stimulated Rock Volume: An Eagle Ford Example. *Unconv. Resour. Technol. Conf.* 24–26 (2017). doi:10.15530/urtec-20172670034
113. Wang, H. F. *et al.* In-Situ Stress Measurement at 1550-meters depth at the kISMET test site in Lead, SD. in *51st US Rock Mechanics/Geomechanics Symposium* 1–7 (2017).
114. Kuang, W., Zoback, M. D. & Zhang, J. Estimating geomechanical parameters from microseismic plane focal mechanisms recorded during multistage hydraulic fracturing. *Geophysics* **82**, KS1–KS11 (2017).
115. Yoon, C. E., Huang, Y., Ellsworth, W. L. & Beroza, G. C. Seismicity During the Initial Stages of the Guy-Greenbrier, Arkansas, Earthquake Sequence. *J. Geophys. Res. Solid Earth* **122**, 9253–9274 (2017).
